# Leukoaraiosis Is Not Associated With Recovery From Aphasia in the First Year After Stroke

**DOI:** 10.1162/nol_a_00115

**Published:** 2023-10-31

**Authors:** Alexandra C. Brito, Deborah F. Levy, Sarah M. Schneck, Jillian L. Entrup, Caitlin F. Onuscheck, Marianne Casilio, Michael de Riesthal, L. Taylor Davis, Stephen M. Wilson

**Affiliations:** School of Medicine, Vanderbilt University, Nashville, TN, USA; Department of Hearing and Speech Sciences, Vanderbilt University Medical Center, Nashville, TN, USA; Department of Radiology, Vanderbilt University Medical Center, Nashville, TN, USA; School of Health and Rehabilitation Sciences, University of Queensland, Brisbane, Australia

**Keywords:** aphasia, brain health, cerebral small vessel disease, leukoaraiosis, stroke

## Abstract

After a stroke, individuals with aphasia often recover to a certain extent over time. This recovery process may be dependent on the health of surviving brain regions. Leukoaraiosis (white matter hyperintensities on MRI reflecting cerebral small vessel disease) is one indication of compromised brain health and is associated with cognitive and motor impairment. Previous studies have suggested that leukoaraiosis may be a clinically relevant predictor of aphasia outcomes and recovery, although findings have been inconsistent. We investigated the relationship between leukoaraiosis and aphasia in the first year after stroke. We recruited 267 patients with acute left hemispheric stroke and coincident fluid attenuated inversion recovery MRI. Patients were evaluated for aphasia within 5 days of stroke, and 174 patients presented with aphasia acutely. Of these, 84 patients were evaluated at ∼3 months post-stroke or later to assess longer-term speech and language outcomes. Multivariable regression models were fit to the data to identify any relationships between leukoaraiosis and initial aphasia severity, extent of recovery, or longer-term aphasia severity. We found that leukoaraiosis was present to varying degrees in 90% of patients. However, leukoaraiosis did not predict initial aphasia severity, aphasia recovery, or longer-term aphasia severity. The lack of any relationship between leukoaraiosis severity and aphasia recovery may reflect the anatomical distribution of cerebral small vessel disease, which is largely medial to the white matter pathways that are critical for speech and language function.

## INTRODUCTION

Aphasia, an acquired impairment of language, is a common and debilitating consequence of many left hemispheric strokes. Frequently, patients with aphasia recover language function over time to varying extents. Prior work investigating the course of language recovery demonstrated that the most rapid gains in speech and language function are made in the first year after stroke (Kertesz & McCabe, 1977; Pedersen et al., 1995; Swinburn et al., 2004; Wilson et al., 2023). A wide range of patient-related and stroke-related factors have been evaluated as predictors of aphasia recovery, with lesion location and extent proving most influential (Basso, 1992; Plowman et al., 2012; Watila & Balarabe, 2015; Wilson et al., 2023). Aphasia recovery is thought to be driven by neural plasticity, that is, the functional reorganization of surviving brain regions such that they take on new or enhanced roles in language processing (Hartwigsen & Saur, 2019; Wilson & Schneck, 2021). The potential for neuroplastic recovery from aphasia may depend in part on the health of surviving brain regions (Basilakos et al., 2019; Johnson et al., 2022; Varkanitsa et al., 2020; Wright et al., 2018).

A major contributor to brain health is cerebral small vessel disease (cSVD), which is a leading cause of cognitive decline and functional impairment in older adults (Pantoni, 2010; ter Telgte et al., 2018). Neuroimaging biomarkers associated with cSVD include leukoaraiosis, lacunar infarcts, and microhemorrhages. Leukoaraiosis refers to diffuse bilateral and symmetrical white matter abnormalities localized to periventricular and subcortical white matter that are hyperintense on fluid attenuated inversion recovery (FLAIR) magnetic resonance imaging (MRI) and hypodense on computed tomography (CT). Leukoaraiosis is very common in older individuals, and is associated with age, hypertension, and other cardiovascular risk factors (de Leeuw et al., 2001; Longstreth et al., 1996). The clinical findings associated with leukoaraiosis include cognitive decline (Au et al., 2006; de Groot et al., 2000), depression (Teodorczuk et al., 2010), apathy (Hollocks et al., 2015), gait disturbances (Baezner et al., 2008), poorer stroke outcomes (Arsava et al., 2009; Kissela et al., 2009; Ryu et al., 2017), and increased risk of stroke and dementia (Debette et al., 2010; Debette & Markus, 2010).

While the association between leukoaraiosis and cognition is robust, only a few studies have investigated whether leukoaraiosis is predictive of recovery from aphasia after stroke. One recent well-powered study found that leukoaraiosis contributes to aphasia severity in chronic stroke patients (Johnson et al., 2022). Several smaller studies have reported associations between leukoaraiosis and naming (Wright et al., 2018), aphasia severity (Wilmskoetter et al., 2019), longitudinal change in aphasia severity in the chronic post-stroke period (Basilakos et al., 2019), and improvement on trained items in the context of aphasia treatment (Varkanitsa et al., 2020). Only one study has investigated the effect of leukoaraiosis on the presence of acute aphasia, finding no relationship (Flowers et al., 2017). No study to our knowledge has explored whether leukoaraiosis is associated with the extent of recovery from aphasia in the early post-stroke period. This phase is particularly important to investigate, because the majority of behavioral gains take place in the first year after stroke (Kertesz & McCabe, 1977; Pedersen et al., 1995; Swinburn et al., 2004; Wilson et al., 2023).

To explore the hypothesis that recovery from aphasia depends on the health of surviving brain regions, we examined leukoaraiosis as a predictor of initial aphasia severity, aphasia recovery, and longer-term aphasia severity in the context of a large prospective study of individuals recovering from aphasia after stroke. We hypothesized that more extensive leukoaraiosis might be associated with more severe aphasia in the acute phase, less recovery over time, and/or poorer longer-term aphasia outcomes. A better understanding of the factors that contribute to recovery from aphasia will enhance the accuracy of prognoses and may provide clues as to how recovery can be maximized.

## METHODS

This study is one component of a prospective longitudinal study of the neural correlates of recovery from aphasia; see Wilson et al. (2023) for additional methodological details. Our study was conducted in accordance with the principles of the 1964 Declaration of Helsinki and was approved by the Institutional Review Board at Vanderbilt University Medical Center. All patients or their legally authorized representatives provided written informed consent to participate in the study.

### Participants

We considered for inclusion all patients presenting at Vanderbilt University Medical Center between late 2016 and early 2020. Our inclusion criteria were: (1) acute ischemic or intracerebral hemorrhagic stroke predominantly confined to left hemisphere supratentorial regions, or right hemisphere stroke with aphasia clearly indicating right hemisphere language dominance; (2) age 18–90 years; and (3) infarct at least 1 cm^3^ except (i) thalamic infarcts were included regardless of extent; (ii) starting after ∼21 months of data collection, basal ganglia and/or subcortical white matter infarcts were included only if they exceeded ∼6 cm^3^. Our exclusion criteria were: (1) unconscious with grave prognosis; (2) not fluent in English premorbidly, as determined by interview with the patient and/or caregiver; (3) prior symptomatic stroke significantly impacting language regions or homotopic regions, neurodegenerative disease, or any other neurological condition impacting language or cognition; (4) major psychiatric disorder; and (5) substance abuse serious enough to interfere with study participation.

**Figure 1.**
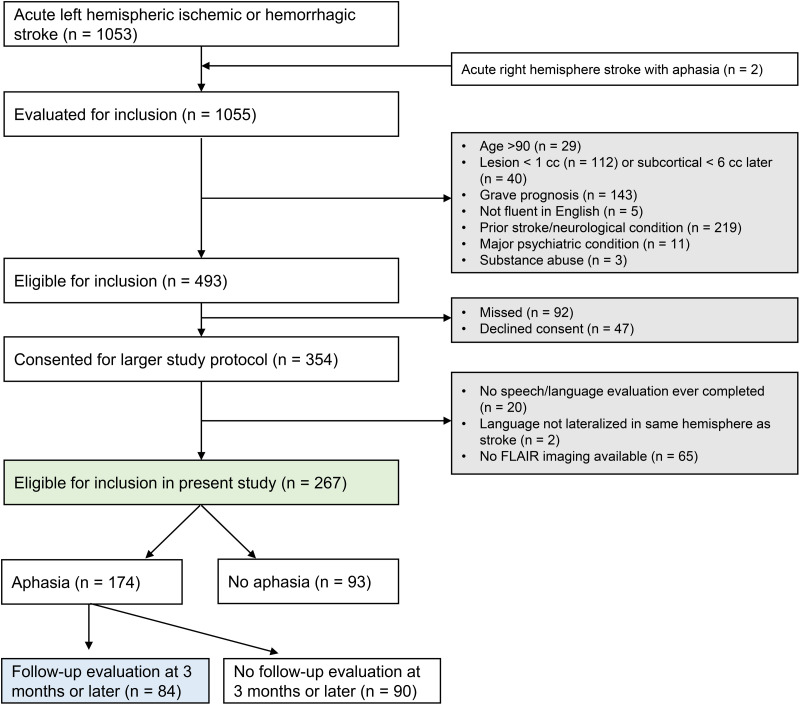
Consort diagram. The green shading signifies patients included in the first analyses (i.e., initial aphasia severity). The blue shading indicates patients included in the second and third analyses (i.e., patients with longitudinal follow-up). FLAIR = fluid attenuated inversion recovery.

As detailed in Figure 1, 1,055 patients met the first inclusion criterion and were evaluated for inclusion, and ultimately 354 met all criteria and consented to participate. For the present analysis of leukoaraiosis in relation to aphasia outcomes, we additionally required (1) at least one speech/language evaluation; (2) availability of acute FLAIR imaging; and (3) language lateralized to stroke hemisphere. A total of 20 patients had no speech/language evaluation, 65 did not have FLAIR imaging (most of these had only CT), and 2 patients had left hemisphere strokes but strong evidence for right hemisphere language dominance, leaving 267 included participants. Demographic and key medical history data for the included patients are shown in Table 1.

**Table 1.** Participant demographics.

	**All participants**	**Participants with longitudinal follow-up**
**Number of participants, *n***	267	84
**Age in years, mean (*SD*)**	62.6 (14.3)	62.5 (13.4)
**Female, *n* (%)**	138 (51.7%)	38 (45.2%)
**Handedness**		
Right-handed, *n* (%)	234 (87.6%)	72 (85.7%)
Left-handed or ambidextrous, *n* (%)	33 (12.4%)	12 (14.3%)
**Race**		
Black, *n* (%)	35 (13.6%)	9 (10.7%)
White, *n* (%)	231 (86.5%)	74 (88.1%)
Other, *n* (%)	1 (0.4%)	1 (1.2%)
**Education in years, mean (*SD*)**	13.1 (3.1)	13.3 (2.9)
**Comorbidities**		
Hypertension, *n* (%)	205 (76.8%)	64 (76.2%)
Diabetes mellitus, *n* (%)	76 (28.4%)	25 (29.8%)
Hyperlipidemia, *n* (%)	147 (55.1%)	53 (63.1%)
Tobacco use (current or former), *n* (%)	148 (55.4%)	50 (59.5%)
**Lesion extent in cm^3^, mean (*SD*)**	40.2 (56.2)	69.1 (77.8)
**Damage to core language areas in cm^3^, mean (*SD*)**	9.2 (15.9)	17.3 (22.0)
NIHSS on admission, mean (*SD*)*	7.7 (7.2)	9.0 (7.2)
**Stroke subtype**		
Hemorrhagic, *n* (%)	33 (12.4%)	11 (12.9%)
Cardioembolic, *n* (%)	84 (31.5%)	28 (32.9%)
Large vessel, *n* (%)	50 (18.7%)	15 (17.6%)
Small vessel, *n* (%)	15 (5.6%)	2 (2.4%)
Other/undetermined etiology, *n* (%)	85 (31.8%)	28 (32.9%)
**Hemorrhagic transformation**		
Yes, *n* (%)	31 (11.6%)	10 (11.8%)
No, *n* (%)	236 (88.4%)	74 (87.1%)

*Note*. *NIHSS scores missing on chart review for 31 patients in the overall patient group and for 12 patients with longitudinal follow-up. NIHSS = National Institutes of Health Stroke Scale.

### Aphasia Assessment

Speech and language function were initially assessed at the bedside within the first 5 days after stroke, on mean day 2.7 (*SD* = 1.3, range = 0, 5). We used the Quick Aphasia Battery (QAB), which is a time-efficient aphasia assessment that has been validated in the acute care context (Wilson, Eriksson, et al., 2018). The QAB shows excellent sensitivity, specificity, test–retest reliability, and concurrent validity with respect to the Western Aphasia Battery (WAB), and is sensitive to change in the acute period and beyond (Wilson et al., 2019; Wilson et al., 2023). Aphasia severity was quantified with the QAB overall score, which ranges from 0 to 10 with 10 representing a perfect score and 0 reflecting the most severe aphasia; patients who were untestable were also scored as 0. The overall score reflects performance on multiple speech and language domains including word and sentence comprehension, word finding, grammatical construction, phonological encoding, speech motor programming, and reading aloud. All language evaluations were administered by certified speech-language pathologists (SMS, JLE, CFO). Audio and video recordings were made of all sessions, which were transcribed and scored offline. All scores were reviewed in consensus meetings of from four to six of the authors.

For all patients who presented with acute aphasia per clinical impression, we then attempted to obtain additional speech-language evaluations at ∼1 month, ∼3 months and ∼12 months. Note that we considered clinical impression to be the gold standard for aphasia diagnosis and that some patients with aphasia per clinical impression scored above the QAB cutoff for aphasia (Wilson, Eriksson, et al., 2018). Most follow-up evaluations were performed at Vanderbilt University Medical Center, inpatient rehabilitation facilities, or patients’ homes. Some of the final time point evaluations were obtained considerably later than 12 months due to the COVID-19 pandemic. For the present study, we were concerned with longer term aphasia outcomes, so we included in our longitudinal analyses all patients who were seen at the ∼3 month time point or later; for each patient we used the latest time point available. The rationale for this was that language recovery was relatively modest between 3 months and 12 months (Wilson et al., 2023), and it was straightforward to include in models the time post onset that the final speech-language evaluation was obtained. There were 174 patients with initial aphasia, of whom 84 were included in the analysis of longer-term outcomes. These longer-term evaluations were acquired on mean day 315 (*SD* = 163, range = 85, 665).

Information regarding the amount of speech-language therapy received between discharge from acute care and the final assessment was available for 76 out of 84 patients: Patients received a mean of 54.7 min of treatment per week (*SD* = 57.7, range = 0, 215); note that 20 patients reported receiving no treatment.

### Evaluation of Leukoaraiosis

Acute FLAIR images obtained in the course of routine clinical care were evaluated for degree of leukoaraiosis on acute MRI scans using the Fazekas visual rating scale (Fazekas et al., 1987). There are several techniques for assessing leukoaraiosis by visual inspection. A meta-analysis of the six most commonly used rating scales showed an overall high correlation among all rating scales and no benefit of using one scale over another (Pantoni et al., 2002). We decided to use the Fazekas scale because it is most commonly used clinically and because it takes into account both periventricular and deep white matter hyperintensities, which are scored separately.

**Figure 2.**
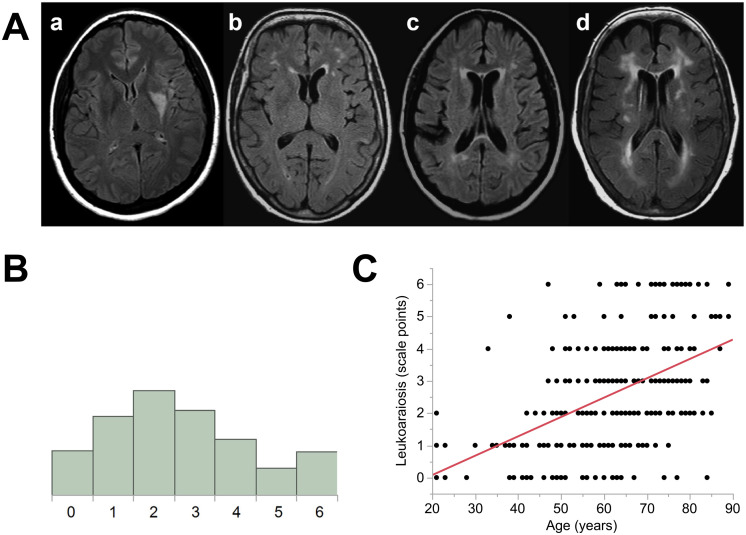
Leukoaraiosis in participant data. (A) Range of leukoaraiosis severity. Written as [periventricular-deep]. a = [0–0], b = [1–1], c = [2–2], d = [3–3]. (B) Distribution of leukoaraiosis ratings. (C) Correlation between age and leukoaraiosis ratings for all patients.

In the Fazekas scale, periventricular and deep white matter hyperintensities are rated on a scale from 0 to 3, which are summed for a composite score ranging from 0 to 6. For periventricular ratings, the following criteria were used: 0 = no lesions, 1 = caps or thin line, 2 = smooth halo around ventricles, 3 = extension into the white matter. For deep white matter hyperintensities, we scored as follows: 0 = no lesions, 1 = punctate foci, 2 = beginning confluence of foci, 3 = large confluent areas. See Figure 2A for four representative MRI scans.

Raters were members of the research team (ACB, DFL), who were trained on the assessment of leukoaraiosis by a neuroradiologist (LTD). They were blinded to all clinical information besides the MR images. Leukoaraiosis was examined in both hemispheres, except that hyperintensities in the immediate vicinity of the infarct were not interpreted as leukoaraiosis.

Initial ratings from the two raters exhibited excellent inter-rater reliability (Cicchetti, 1994), with an inter-class correlation coefficient of 0.83 (type A-1). All discrepancies in ratings were resolved by discussion between the two raters, in consultation with the neuroradiologist as necessary, to derive final ratings that were used in subsequent analyses.

### Lesion Mapping

Lesion location and extent are the most important determinants of aphasia outcome. To include these factors in our models, stroke lesions were manually delineated on acute clinical imaging and normalized to standard space (Wilson et al., 2023). Lesion extent was calculated in all of dominant hemisphere supratentorial cortex. Damage to key language areas was quantified as lesion extent within regions activated by either of two language mapping paradigms, one using a semantic decision task and the other using a phonological decision task (Wilson, Yen, & Eriksson, 2018; Yen et al., 2019). These functional maps were based on 16 neurologically normal individuals (Yen et al., 2019) and were thresholded at a voxelwise threshold of *p* < 0.005, then corrected at *p* < 0.05 based on cluster extent using permutation analysis. The language areas identified included the inferior frontal gyrus, ventral precentral gyrus, superior temporal sulcus, middle temporal gyrus, posterior inferior temporal gyrus, supramarginal gyrus, and midline superior frontal gyrus (Yen et al., 2019).

### Statistical Analysis

Three linear models were constructed in order to evaluate the effect of leukoaraiosis on (1) initial QAB scores, (2) aphasia recovery, and (3) longer-term QAB scores. Our statistical approach was closely based on Wilson et al. (2023). All three models included leukoaraiosis as the independent variable of interest, along with the following covariates: age, sex, handedness, education, stroke type (ischemic/hemorrhagic), lesion extent, and lesion extent in language areas. The last two of these covariates were modeled as polynomials of degree 2 since their effects are likely to be nonlinear.

The model of aphasia recovery included two additional covariates known to be predictive of recovery: initial QAB score and days post stroke of the final evaluation, both modeled as polynomials of degree 2 due to expected nonlinear effects. The dependent variable was transformed with an inverse hyperbolic sine function (*λ* = 4) to reduce heteroscedasticity, since lower starting scores entail more room for improvement, so there was more variance when starting scores were lower. The model of final QAB scores also included days post stroke of the final evaluation, as a polynomial of degree 2.

Statistical significance of single terms was assessed using *t* statistics, while statistical significance of sets of related terms was assessed with likelihood ratio tests comparing full and reduced models. One-tailed *p* values are reported for measures of brain health (leukoaraiosis, age) because there were directional hypotheses associated with these measures, while two-tailed *p* values are reported for all other variables.

## RESULTS

### Leukoaraiosis

Evidence of at least some leukoaraiosis was present on 240 out of 267 (90%) FLAIR images. The mean leukoaraiosis rating was 2.6 points (*SD* = 1.7, range = 0, 6), on the Fazekas scale. The distribution of leukoaraiosis ratings is shown in Figure 2B. As expected, leukoaraiosis was highly correlated with age, *r* = 0.50, *p* < 0.0001 (Figure 2C). A linear model of leukoaraiosis as a function of demographic and medical history variables and stroke type showed that there was a medium sized effect of age, *f*^2^ = 0.271, *β* = 0.058 Fazekas points per year, 95% CI = 0.044, 0.072, *p* < 0.0001; a small effect of hypertension, *f*^2^ = 0.034, *β* = 0.696 Fazekas points, 95% CI = 0.231, 1.162, *p* = 0.0035, and a small effect of hemorrhagic stroke, *f*^2^ = 0.036, *β* = 0.848 Fazekas points, 95% CI = 0.297, 1.398, *p* = 0.0027. No other variables were significant predictors of leukoaraiosis.

### Aphasia

At the acute time point, among individuals with and without aphasia, the mean initial QAB overall score was 6.8 (*SD* = 3.1, range = 0, 10). In the 84 patients with aphasia who were included in the longitudinal analyses, the mean initial QAB overall score was 5.1 (*SD* = 0.3, range = 0, 9.75). These patients recovered by a mean of 2.9 points (*SD* = 2.2, range 0, 9.1), obtaining a mean longer term QAB overall score of 8.0 (*SD* = 2.1, range = 0.7, 9.9).

### Effect of Leukoaraiosis on Initial Aphasia Severity

**Figure 3.**
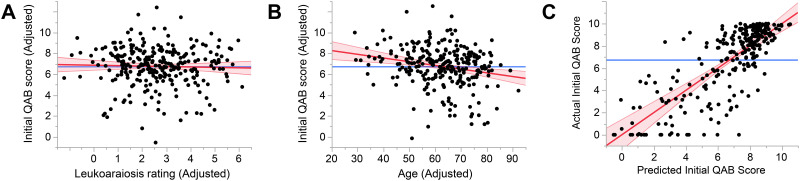
Predictors of initial aphasia severity. (A) Added variable plot (partial regression residual leverage plot) showing the lack of relationship between adjusted leukoaraiosis rating and adjusted initial QAB score. Note that each variable was adjusted by accounting for the effects of other variables in the model, which is why adjusted scores could fall outside the actual range of the scales. (B) Added variable plot showing a significant negative relationship between age and initial QAB score. (C) Whole model plot showing the predictive power of the model. QAB = Quick Aphasia Battery.

There was no effect of leukoaraiosis on initial aphasia severity, *n* = 267, Cohen’s *f*^2^ = 0.0006, *β* = −0.03 QAB points per Fazekas scale point, 95% CI = −0.19, 0.13, *p* = 0.35 (Figure 3A). There was a small negative effect of age, *f*^2^ = 0.045, *β* = −0.033 QAB points per year, 95% CI = −0.052, −0.014, *p* = 0.0004 (Figure 3B). The full model explained 63.8% of the variance, with significant effects (besides age) of total lesion extent, damage to the language network, stroke type, and education (Figure 3C; see Supplemental Table 1 in the Supporting Information, available at https://doi.org/10.1162/nol_a_00115, for details). When periventricular and deep white matter involvement were modeled separately, neither were associated with initial aphasia severity, periventricular: *β* = −0.035, *p* = 0.41; deep: *β* = −0.069, *p* = 0.32. In the subset of 234 patients with ischemic stroke, there was no effect of leukoaraiosis on initial aphasia severity, *β* = −0.078, *p* = 0.18.

Because of the observed correlation between leukoaraiosis and age, we also ran a follow-up model in which age was omitted. In this model, there was a small negative effect of leukoaraiosis on initial aphasia severity, *f*^2^ = 0.02, *β* = −0.17 QAB points per Fazekas scale point, 95% CI = −0.31, −0.03, *p* = 0.0088. However, the fact that leukoaraiosis had no effect in the full model indicates that this effect is secondary to the correlation between leukoaraiosis and age.

### Effect of Leukoaraiosis on Recovery From Aphasia

**Figure 4.**
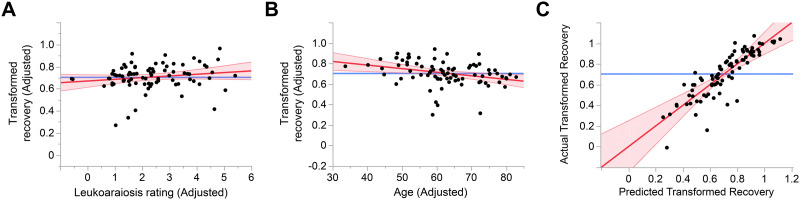
Predictors of recovery from aphasia. (A) Added variable plot (partial regression residual leverage plot) showing the lack of relationship between adjusted leukoaraiosis rating and adjusted transformed recovery. (B) Added variable plot showing a significant negative relationship between age and transformed recovery. (C) Whole model plot showing the predictive power of the model.

There was no effect of leukoaraiosis on recovery from aphasia (the extent of improvement in QAB overall score from the acute time point to the longer-term time point), *n* = 84, Cohen’s *f*^2^ = 0.03, *β* = 0.016 transformed QAB points per Fazekas scale point, that is, numerically greater recovery in patients with worse leukoaraiosis, 95% CI = −0.006, 0.037, *p* = 0.93 (Figure 4A). There was a small negative effect of age on recovery, *f*^2^ = 0.11, *β* = −0.0035 transformed QAB points per year, 95% CI = −0.0060, −0.0009, *p* = 0.0039 (Figure 4B). The full model explained 75.0% of the variance, with significant effects (besides age) of initial aphasia severity, total lesion extent, damage to the language network, and handedness (Figure 4C; see Supplemental Table 2 for details).

When periventricular and deep white matter involvement were modeled separately, neither were associated with recovery from aphasia, periventricular: *β* = 0.043, *p* = 0.99; deep: *β* = −0.0075, *p* = 0.65, nor was leukoaraiosis associated with recovery in the subset of 73 patients with ischemic stroke, *β* = 0.020, *p* = 0.94. There was no effect of leukoaraiosis in a follow-up model with age omitted.

### Effect of Leukoaraiosis on Longer-Term Aphasia Outcome

**Figure 5.**
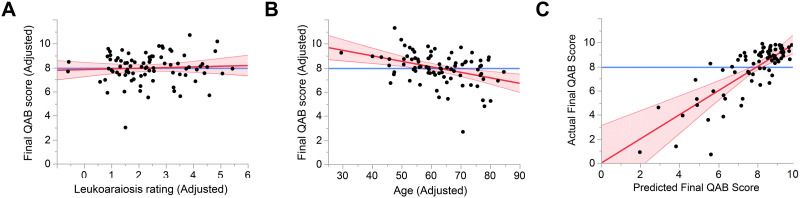
Predictors of longer-term aphasia outcome. (A) Added variable plot (partial regression residual leverage plot) showing the lack of relationship between adjusted leukoaraiosis ratings and adjusted final QAB score. (B) Added variable plot showing a significant negative relationship between age and adjusted final QAB score. (C) Whole model plot showing the predictive power of the model. QAB = Quick Aphasia Battery.

There was no effect of leukoaraiosis on longer-term aphasia outcome (QAB overall score at ∼3 months or later), *n* = 84, Cohen’s *f*^2^ = 0.004, *β* = 0.062 QAB points per Fazekas scale point, that is, numerically better outcomes in patients with worse leukoaraiosis, 95% CI = −0.166, 0.290, *p* = 0.70 (Figure 5A). There was a medium-sized negative effect of age on final aphasia outcome, *f*^2^ = 0.17, *β* = −0.045 QAB points per year, 95% CI = −0.071, −0.019, *p* = 0.0005 (Figure 5B). The full model explained 66.1% of the variance, with significant effects (besides age) of total lesion extent and damage to the language network (Figure 5C; see Supplemental Table 3 for details).

When periventricular and deep white matter involvement were modeled separately, neither were associated with longer term aphasia outcome, periventricular: *β* = 0.147, *p* = 0.76; deep: *β* = 0.049, *p* = 0.40, nor was leukoaraiosis associated with outcome in the subset of 73 patients with ischemic stroke, *β* = 0.075, *p* = 0.74. There was no effect of leukoaraiosis in a follow-up model with age omitted.

## DISCUSSION

In this study, we evaluated the impact of leukoaraiosis on three outcomes of interest: initial aphasia severity, aphasia recovery, and longer-term aphasia severity. We observed no significant associations between baseline leukoaraiosis severity, as measured by the Fazekas visual rating scale, and any of these outcome measures. Strengths of our study include its prospective and longitudinal design, representative sample of ischemic and hemorrhagic stroke patients, reliable quantification of leukoaraiosis, and careful quantification of aphasia using a validated battery. The confidence intervals around the estimates of the effect of leukoaraiosis on these three outcome measures were quite narrow, indicating that any effect of leukoaraiosis is at most modest. Moreover, our study was sufficiently well powered to demonstrate robust negative effects of age on aphasia outcomes.

**Figure 6.**
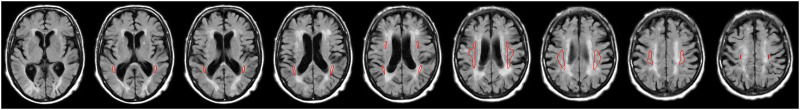
The superior longitudinal fasciculus (from the JHU atlas, Hua et al., 2008) overlaid on a FLAIR image from an individual with severe leukoaraiosis. FLAIR = fluid attenuated inversion recovery.

There is strong and unequivocal evidence that leukoaraiosis is associated with cognitive deficits and cognitive decline (Au et al., 2006; de Groot et al., 2000). Why then, would leukoaraiosis not show an association with language deficits or their resolution? One possible explanation is that language is anatomically and functionally distinct from other aspects of cognition (Blank et al., 2014; Fedorenko et al., 2012; Fedorenko & Varley, 2016; Woolgar et al., 2018). The anatomical distribution of leukoaraiosis is relatively conserved across individuals: primarily periventricular and particularly concentrated around the frontal and occipital horns of the ventricles. This characteristic distribution of cSVD is largely medial to critical language tracts, which may explain the lack of association between leukoaraiosis and aphasia recovery that we observed. Figure 6 shows the location of the superior longitudinal fasciculus, the most dorsal of the white matter pathways that are critical for language (Wilson et al., 2011), in relation to the distribution of white matter hyperintensities in an individual with severe periventricular and deep white matter leukoaraiosis. This illustrates how even severe leukoaraiosis impinges only minimally on the language network.

However, this potential explanation for our null findings should be considered tentative, because cSVD has been shown to have network effects well beyond its visible markers (leukoaraiosis, microbleeds, enlarged perivascular spaces, etc.; ter Telgte et al., 2018; Wilmskoetter et al., 2019). These more global effects may include disruption of white matter pathways, abnormal functional connectivity, and atrophy. Therefore, the fact that leukoaraiosis is largely localized medial to the language network does not imply that cSVD spares the language network entirely.

Several previous studies have investigated the relationship between leukoaraiosis and aphasia. The largest and most comprehensive of these reported that, in contrast to our findings, severity of leukoaraiosis was negatively associated with aphasia outcome in 106 chronic stroke patients (Johnson et al., 2022; see also two previous smaller studies from the same group: Basilakos et al., 2019; Wilmskoetter et al., 2019). We calculated the effect size of leukoaraiosis based on the data set provided (Johnson et al., 2022) and found that the effect size of the association between leukoaraiosis and aphasia was small, Cohen’s *f*^2^ = 0.06, *β* = −2.8 WAB points per Fazekas scale point, 95% CI = −5.1, −0.5. Note that the WAB is on a 100-point scale in contrast to the 10-point scale of the QAB in the present study.

One potentially important difference between this study and ours is that in Johnson et al. (2022), patients were recruited because they expressed an interest in taking part in aphasia treatment studies, and as such they generally had significant chronic aphasia. The mean WAB aphasia quotient was 62.2 and the mean lesion extent was 121 cm^3^, indicating that this cohort was considerably more severe than our longitudinal cohort, who had recovered to a mean QAB overall score of 8.0 by our final timepoint, and had a mean lesion extent of 69 cm^3^. It is possible that leukoaraiosis is more of a predictor of recovery in patients with the kind of substantial damage to core language regions that leads to chronic moderate to severe aphasia, but less of a factor for the majority of left hemisphere stroke patients, who tend to recover quite well (Wilson et al., 2023).

Another possibility is that leukoaraiosis modulates treated recovery but not spontaneous recovery. This would be plausible because many forms of aphasia treatment draw on cognitive, attentional, and executive processes that are distinct from the language network, and these are the kinds of cognitive processes that have been shown to be impacted by leukoaraiosis. The patients in Johnson et al. (2022) had mostly received substantial aphasia treatment, and their mean time post stroke was just over 3 years, so these patients had spent a longer period of time in the recovery process. In contrast, our cohort of patients were a more representative sample of left hemisphere stroke survivors, who had mostly received relatively low intensity speech-language therapy, or none at all, so their recovery was largely spontaneous in nature.

Two other smaller studies have reported associations between leukoaraiosis and aphasia. In 42 patients who had presented with aphasia acutely after stroke, leukoaraiosis was associated with naming and verbal fluency measures when patients were assessed later in the subacute or chronic phase (Wright et al., 2018). Besides its small sample size, this study had several methodological limitations including ceiling effects on the naming measure, dichotomization of outcomes, and failure to include age as a covariate. In the other study, leukoaraiosis was associated with an improvement on trained items in the context of aphasia treatment (Varkanitsa et al., 2020). This positive finding may again reflect the impact of leukoaraiosis on the cognitive abilities that underpin successful response to treatment, though it was not reported whether leukoaraiosis had any impact on generalization to untrained items, or other measures of aphasia severity. Finally, one previous study of the association between leukoaraiosis and acute aphasia did not find any relationship (Flowers et al., 2017). This result is consistent with our findings; however, this study had several limitations including binary diagnoses of aphasia and a narrow range of leukoaraiosis scores for most of the patients.

Our study had several noteworthy limitations. First, although we had a large sample of acute stroke patients (*N* = 267), our sample of patients with longer term speech-language evaluations was smaller (*n* = 84). It remains possible that there is a small effect of leukoaraiosis on recovery from aphasia that we did not detect in our sample. Furthermore, we did not study patients who were missed in the hospital (e.g., due to rapid discharge), who declined consent, or for whom FLAIR imaging was not acquired. Second, the Fazekas scale is a subjective visual rating scale. While this scale is widely used clinically and has high inter-rater reliability, future work would benefit from a volumetric assessment of leukoaraiosis. Third, we focused only on leukoaraiosis and did not investigate other potential measures of brain health, such as atrophy, microhemorrhages, or lacunar strokes.

Despite these limitations, our study demonstrates that leukoaraiosis likely is not a major contributor to language outcomes after stroke, either acutely or throughout the first year, during which the majority of behavioral gains take place. The major determinants of aphasia after stroke are lesion location and extent (Wilson et al., 2023). Age has a modest negative effect on outcomes, though what aspect(s) of aging are responsible for this effect remain unknown. Despite our negative findings for leukoaraiosis in this study, it remains plausible that the health of surviving brain regions is a contributing factor to unexplained variance in aphasia recovery. Further research is needed to discover whether there may be other imaging biomarkers that may capture the variability of surviving brain regions in their potential for neuroplasticity.

## ACKNOWLEDGMENTS

The authors thank Annie Burchfield, Wayneho Kam, Howard Kirshner, Maysaa Rahman, Emma Willey, Melodie Yen, two anonymous reviewers, and the many stroke survivors, caregivers, and clinicians who helped make the study possible.

## FUNDING INFORMATION

Stephen M. Wilson, National Institute on Deafness and Other Communication Disorders (https://dx.doi.org/10.13039/100000055), Award ID: R01 DC013270.

## AUTHOR CONTRIBUTIONS

**Alexandra C. Brito**: Conceptualization; Formal analysis; Investigation; Visualization; Writing – original draft; Writing – review & editing. **Deborah F. Levy**: Investigation; Writing – review & editing. **Sarah M. Schneck**: Investigation; Writing – review & editing. **Jillian L. Entrup**: Investigation; Writing – review & editing. **Caitlin F. Onuscheck**: Investigation; Writing – review & editing. **Marianne Casilio**: Investigation; Writing – review & editing. **Michael de Riesthal**: Supervision; Writing – review & editing. **L. Taylor Davis**: Investigation; Supervision; Writing – review & editing. **Stephen M. Wilson**: Conceptualization; Formal analysis; Funding acquisition; Methodology; Supervision; Writing – original draft; Writing – review & editing.

## DATA AVAILABILITY STATEMENT

The data and code for the key analyses described are available at: https://doi.org/10.17605/OSF.IO/D45CF.
