# Supplementary material for: Leukoaraiosis Is Not Associated With Recovery From Aphasia in the First Year After Stroke
Source: Neurobiol Lang (Camb). 2023 Oct 31;4(4):536–49. doi: 10.1162/nol_a_00115 (PMC10631799; doi:10.1162/nol_a_00115)
Supplement: Supplementary file 1 [file nol-4-4-536-s001.pdf]

Supplemental Material for Brito et al., 'Leukoaraiosis is not associated with recovery from aphasia in the first year after stroke'

Supplemental Table 1 Linear model of initial aphasia severity (QAB overall score)

	ß	95% CI		*p*
Intercept	9.424	7.785	11.063	< .0001
Leukoaraiosis (Fazekas scale points)	−0.032	−0.189	0.126	.69
Age (years)	−0.0332	−0.052	−0.0140	.0008
Sex (female)	0.463	−0.00081	0.927	.050
Handedness (non-right-handed)	0.171	−0.538	0.879	.64
Education (years)	0.104	0.0289	0.180	.0069
Stroke type (hemorrhagic)	−1.359	−2.094	−0.625	.0003
Lesion extent (cm^3)	−0.0214	−0.041	−0.0013	.0006
Lesion extent (cm^3, squared)	−9.58e−06	−0.00010	8.58e−05	
Damage to core language areas (cm^3)	−0.200	−0.277	−0.123	< .0001
Damage to core language areas (cm^3, squared)	0.0023	0.00086	0.0038	

Number of observations = 267; Error degrees of freedom = 256; r^2 = 0.638

Supplemental Table 2 Linear model of recovery from aphasia (Transformed QAB points)

	ß	95% CI		*p*
Intercept	1.143	0.868	1.418	< .0001
Leukoaraiosis (Fazekas scale points)	0.016	−0.0058	0.037	.15
Age (years)	−0.0035	−0.0060	−0.00095	.0078
Sex (female)	−0.032	−0.091	0.027	.28
Handedness (non-right-handed)	−0.110	−0.193	−0.027	.010
Education (years)	0.0013	−0.0088	0.011	.80
Stroke type (hemorrhagic)	0.043	−0.049	0.136	.35
Lesion extent (cm^3)	−0.0030	−0.0051	−0.00095	.0012
Lesion extent (cm^3, squared)	1.41e−05	5.99e−06	2.23e−05	
Damage to core language areas (cm^3)	0.0079	−0.00023	0.016	.0014
Damage to core language areas (cm^3, squared)	−0.00020	−0.00033	−7.30e−05	
Initial QAB	0.014	−0.023	0.050	< .0001
Initial QAB (squared)	−0.0087	−0.013	−0.0048	
Time post stroke (days)	0.00025	−0.00042	0.00092	.08
Time post stroke (days, squared)	−1.23e−07	−1.20e−06	9.57e−07	

Number of observations = 84; Error degrees of freedom = 69; r^2 = 0.750

Supplemental Table 3 Linear model of longer term aphasia outcome (QAB overall score)

	ß	95% CI		*p*
Intercept	11.445	8.872	14.018	< .0001
Leukoaraiosis (Fazekas scale points)	0.062	−0.166	0.290	.59
Age (years)	−0.045	−0.071	−0.019	.0010
Sex (female)	−0.037	−0.658	0.585	.91
Handedness (non-right-handed)	−0.638	−1.496	0.221	.14
Education (years)	0.014	−0.092	0.119	.80
Stroke type (hemorrhagic)	−0.221	−1.154	0.713	.64
Lesion extent (cm^3)	−0.030	−0.052	−0.0081	.0076
Lesion extent (cm^3, squared)	0.00012	3.78e−05	0.00021	
Damage to core language areas (cm^3)	0.023	−0.059	0.105	.0009
Damage to core language areas (cm^3, squared)	−0.0016	−0.0029	−0.00023	
Time post stroke (days)	0.0037	−0.0033	0.011	.12
Time post stroke (days, squared)	−3.43e−06	−1.48e−05	7.92e−06	

Number of observations = 84; Error degrees of freedom = 71; r^2 = 0.661
